# *Panax quinquefolius* saponins combined with dual antiplatelet therapy enhanced platelet inhibition with alleviated gastric injury via regulating eicosanoids metabolism

**DOI:** 10.1186/s12906-023-04112-7

**Published:** 2023-08-18

**Authors:** Wenting Wang, Lei Song, Lin Yang, Changkun Li, Yan Ma, Mei Xue, Dazhuo Shi

**Affiliations:** 1 National Clinical Research Center for Chinese Medicine Cardiology, Xiyuan Hospital, China Academy of Chinese Medical Sciences, Beijing, 100091 China; 2 Affiliated Hangzhou Chest Hospital, Zhejiang University School of Medicine, Hangzhou, 310003 China; 3 Center of Cardiovascular Disease, Xiyuan Hospital, China Academy of Chinese Medical Sciences, Beijing, 100091 China; 4 Shimadzu (China) Co., LTD Beijing Branch, Beijing, 100020 China; 5 Department of Pathophysiology and Allergy Research, Center of Pathophysiology, Infectiology & Immunology, Vienna General Hospital, Medical University of Vienna, 1090 Vienna, Austria

**Keywords:** *Panax quinquefolius* saponins, Dual antiplatelet therapy, Platelet inhibition, Gastric injury, Lipidomics, Eicosanoids

## Abstract

**Background:**

*Panax quinquefolius* saponin (PQS) was shown beneficial against platelet adhesion and for gastroprotection. This study aimed to investigate the integrated efficacy of PQS with dual antiplatelet therapy (DAPT) on platelet aggregation, myocardial infarction (MI) expansion and gastric injury in a rat model of acute MI (AMI) and to explore the mechanism regarding arachidonic acid (AA)-derived eicosanoids metabolism.

**Methods:**

Wistar rats were subjected to left coronary artery occlusion to induce AMI model followed by treatment with DAPT, PQS or the combined therapy. Platelet aggregation was measured by light transmission aggregometry. Infarct size, myocardial histopathology was evaluated by TTC and H&E staining, respectively. Gastric mucosal injury was examined by scanning electron microscope (SEM). A comprehensive eicosanoids profile in plasma and gastric mucosa was characterized by liquid chromatography-mass spectrometer-based lipidomic analysis.

**Results:**

PQS+DAPT further decreased platelet aggregation, lessened infarction and attenuated cardiac injury compared with DAPT. Plasma lipidomic analysis revealed significantly increased synthesis of epoxyeicosatrienoic acid (EET) and prostaglandin (PG) I_2_ (potent inhibitors for platelet adhesion and aggregation) while markedly decreased thromboxane (TX) A_2_ (an agonist for platelet activation and thrombosis) by PQS+DAPT, relative to DAPT. DAPT induced overt gastric mucosal damage, which was attenuated by PQS co-administration. Mucosal gastroprotective PGs (PGE_2_, PGD_2_ and PGI_2_) were consistently increased after supplementation of PQS+DAPT.

**Conclusions:**

Collectively, PQS+DAPT showed synergistic effect in platelet inhibition with ameliorated MI expansion partially through upregulation of AA/EET and AA/PGI_2_ synthesis while suppression of AA/TXA_2_ metabolism. PQS attenuated DAPT-induced gastric injury, which was mechanistically linked to increased mucosal PG production.

**Supplementary Information:**

The online version contains supplementary material available at 10.1186/s12906-023-04112-7.

## Background

Irregulated platelet activation plays a crucial role in atherothrombosis. Dual antiplatelet therapy (DAPT) with aspirin (ASA) and clopidogrel (CLP) is the mainstay of secondary prevention in the setting of acute coronary syndromes (ACS) [1]. However, a considerable number of patients exhibit insufficient platelet inhibition despite DAPT [2–4], which has been associated with a higher risk for atherothrombotic events, such as stent thrombosis and myocardial infarction (MI) expansion [5, 6]. ASA induces a wide spectrum of gastrointestinal (GI) complications, ranging from mucosal erosion, ulcer to sever bleeding [7], which may have an adverse impact on prognosis [8]. DAPT poses significantly increased risks of GI injury compared with ASA alone [9]. Therefore, more effective antiplatelet strategies with limited GI toxicity are much needed.

*Panax quinquefolius* (PQ), commonly known as North American ginseng, possesses a wide range of biological effects and has been extensively employed in Asian societies, particularly in the cardiovascular system [10]. *Panax quinquefolius* saponins (PQS), major bioactive component of stem and leaves of PQ, is a patent Chinese herbal medicine approved by National Medical Products Administration (commercial name as Xinyue capsule, Z20030073) for the treatment of coronary artery disease. The pharmacological actions of PQS have been generally ascribed to multiple triterpenoid ginsenosides (Rb1, Rb2, Rc, Rd, Re and Rg1) as evidenced by high-performance liquid chromatography ultraviolet analysis [11]. We previously revealed that PQS combined with DAPT significantly improved inhibitory effect on platelet adhesion to injured human umbilical vein endothelial cells (HUVECs) [12], an initial phase of platelet activation and thrombosis [13]. Additionally, PQS was shown beneficial for reducing DAPT-related gastric injury in vivo [14]. The joint actions of PQS+DAPT were achieved through unresolved mechanisms that may involve regulation of arachidonic acid (AA) pathway.

Eicosanoids arise from the oxidation of AA and other 20 carbon chain polyunsaturated fatty acids by cyclooxygenase (COX), lipoxygenase (LOX), cytochrome P450 (CYP) enzymes, or via nonenzymatic mechanism. Eicosanoids are highly bioactive oxylipins acting on many cell types and exert multitudinous functions in the cardiovascular, renal and GI systems [15]. Specifically, AA-derived eicosanoids have been found to exhibit anti- or pro-thrombotic activity via regulating platelet and endothelial functions [16]. Additionally, the crucial roles of AA-derived eicosanoids in GI mucosal defense, as well as in GI injury, have also been extensively reported [17, 18]. In our previous in vitro study, the more potent anti-adhesion effect of PQS+DAPT, compared with DAPT, was mechanically linked to enhanced synthesis of endothelial prostaglandin (PG) I_2_ [12], an AA/COX downstream eicosanoid with potent antiplatelet action [19]. Interestingly, ameliorated gastric injury by adding PQS to DAPT was associated with AA/COX/PGE_2_ pathway [14]. These results suggested a regulatory role of PQS in AA-derived eicosanoids metabolism, which warrant further investigations.

In the present study, we examined the integrated efficacy of PQS combined with DAPT on platelet inhibition, MI expansion and gastric injury in a rat model of acute MI (AMI). The emerging field of pharmacometabolomics, including lipidomics, have contributed to understanding the mechanism of drug actions through identification of metabolic signatures associated with therapeutic responses [20]. Mechanistically, we conducted an AA-targeted lipidomic analysis of eicosanoids metabolic alterations in response to PQS+DAPT, relative to DAPT, in rat plasma and gastric mucosa.

## Methods

### Drugs and reagents

PQS (commercial name as Xinyue capsules, 50 mg PQS/capsule, National medicine permit No. Z20030072) was provided by Yisheng Pharmaceutical Co., Ltd (Jilin, China, batch No.180102). PQS was shown consistent quality between different batches and six ginsenosides (Rb1, Rb2, Rc, Rd, Re and Rg1) were detected as the major active compounds of PQS by our previous high-performance liquid chromatography ultraviolet analysis (the chemical structure of these ginsenosides were shown in Fig. S1) [11]. ASA was purchased from BAYER (Beijing, China). CLP was purchased from Sanofi (Paris, France). Adenosine diphosphate (ADP) were purchased from Chrono-log Corporation (Havertown, Pennsylvania, USA). 2,3,5-triphenyltetrazolium chloride (TTC) was purchased from Sigma-Aldrich (St. Louis, MO, USA). Internal standards for lipidomics analysis including 6-keto-PGF1α-d4, Thromboxane (TX) B_2_-d4, PGF2a-d4, PGE_2_-d4, PGD_2_-d4, Leukotriene (LT) C_4_-d5, LTB_4_ d4, 15-hydroxyeicosatetraenoic acid (HETE) -d8, 12-HETE-d8, 5-HETE -d8, AA-d8 were purchased from Cayman Chemical (Ann Arbor, Michigan, USA). The liquid chromatography-mass spectrometer (LC-MS)-grade solvents, methyl alcohol, acetonitrile, formic acid and ethanol were purchased from Thermo Fisher Scientific (Waltham, Massachusetts, USA).

### Animals and ethic

All animal protocols were approved by the Xiyuan Hospital Institutional Animal Care and Use Committee and performed in accordance with the national guidelines for the care and use of laboratory animals issued by Ministry of Science and Technology of the People’s Republic of China. This study was reported in accordance with ARRIVE (Animal Research: Reporting of In Vivo Experiments) guidelines. Male Wistar rats (weighted between 180 and 210 g) were purchased from Beijing University Laboratory Animal Center (Beijing, China; certificate number SCXK [Jing] 2011-0004). Rats were allowed to acclimatize under standard care conditions for at least 7 days.

### AMI model and experimental design

AMI was induced by permanent ligation of left anterior descending (LAD) coronary artery as described previously [21]. At the onset of ligation, rats were weighted between 220 and 250 g. AMI rats were randomized into 4 groups: AMI group, PQS group, DAPT group and PQS+DAPT group (*n* = 12 for each group). Another group of rats were subjected to the same operation except for ligation (sham group, *n* = 12). From day one to day twenty-nine after surgery, rats in drug intervention groups were administered daily by oral gavage with PQS at dose of 27 mg/kg, or ASA at loading dose of 27 mg/kg (for only 1 day) and then maintenance dose of 9 mg/kg combined with CLP at loading dose of 27 mg/kg (for only 1 day) and then maintenance dose of 6.75 mg/kg, or PQS added to DAPT, respectively. Rat PQS dosage was 257 mg/ Kg, which was equivalent to 42.85 mg/ Kg daily for humans as manual recommended. Loading and maintenance dosage of DAPT for rats were converted in accordance with human guideline recommendations [1]. For rats in sham and AMI group, same volume of physiological saline was orally administered.

Twenty-four hours after last medication, rats were euthanized under anesthesia (pentobarbital sodium, 30 mg/kg, IP) for exsanguination by abdominal aorta puncture. Whole blood drawn from abdominal aorta were collect in vacutainer tubes containing 3.8% trisodium citrate [22] and EDTA [23] for platelet aggregatory assay and lipidomic analysis, respectively. Hearts were harvested and maintained at -80 °C for further analysis. One cohort (*n* = 10 for each group) was stained by TTC for infarct volume quantification and the other cohort (*n* = 2 for each group) was subjected to hematoxylin and eosin (H&E) staining for myocardium histopathological examination. Evidence from clinical observations suggests that patients on antiplatelet treatment develop endoscopic-confirmed gastric lesions more frequently in the antrum [24]. Mucosal tissues taken from antrum were used for ultrastructure scanning and lipidomic characterization.

### Measurement of platelet aggregation

Platelet aggregation was measured by light transmission aggregometry assay in a 8-channel aggregometer (Pulisheng Instrument Co. Ltd., Shanghai, China) as previously described [25]. 3.8% trisodium citrate-anticoagulated whole blood was centrifuged (260 × g, 10 min) to obtain platelet-rich plasma (PRP). The remaining blood was further centrifuged (760 × g, 10 min) to obtain platelet-poor plasma (PPP). PRP was adjusted to platelet counts of 1 × 10^8^/mL with PPP. Before agonist stimulation, light transmission was adjusted to 100% with PPP and to 0% with PRP. After preincubation with ADP at final concentration of 10 μmol/L, aggregation curve was recorded over 5 min (with 100% aggregation corresponding to 100% light transmission). Maximal platelet aggregation was calculated as percent change in light transmission.

### Myocardial histopathology

Myocardial issue specimens were fixed in 10% formaldehyde and embedded in paraffin. The waxes were sectioned at 5-µm thickness and then deparaffinized and rehydrated. Standard H&E staining was carried out for histopathological examinations. Myocardium structure was observed under a microscope (Olympus, Tokyo, Japan) and analyzed by Image-pro plus 6.0 (Media Cybernetics, Maryland, USA).

### Infarct size quantification

The myocardial infarct size was evaluated by TTC staining as previously described [26]. Briefly, the frozen heart was sectioned into 5 slices along the left ventricle (LV) long axis and stained with 0.25% TTC at 37 °C for 30 min in the dark. The stained heart slices were photographed with a digital camera. The infarct size was visualized using Image J (Bethesda, MD, United States) and quantified according to the equation:

$$[\mathrm{infarct\, area\, of\, LV}/(\mathrm{cross}-\mathrm{sectional\, area\, of\, LV }-\mathrm{ area\, of\, LV\, chamber})] \times 100\mathrm{\%}$$

### Observation of gastric mucosal injury

Gastric antrum mucosal samples (40 × 10 × 10 mm block) were pre-fixed with 2.5% glutaraldehyde (pH = 7.2) at room temperature for 3 days followed by post-fixation with 1% osmium for 2 h at room temperature. Samples were dehydrated in ascending grades of ethanol (30%, 40%, 50%, 60%, 70%, 80%, 90%, 95%, 100%, each for 10 min). Samples were critical point dried and coated with gold palladium. The ultrastructure of gastric mucosa was imaged with a scanning electron microscope (SEM) (HITACHI, Japan) operating at 10 kV.

### Lipidomic analysis

#### Sample preparation and lipid extraction

AA-derived eicosanoids were extracted according to the procedure described previously [27]. EDTA-anticoagulated whole blood was centrifuged (2000 × g, 15 min, 4 °C) and plasma was collected. Internal standards were added into 50 mg of gastric mucosa and 600 μl plasma samples at the final concentration of 83 ng/mL (except for AA-d8 of 166 ng/mL). Samples were then vortexed for 3 min. After centrifugation (4000 × g, 5 min, 4 °C), the upper layer (550 μL of gastric mucosal sample and 1 mL of plasma) was supplemented with 4 mL 0.1% formic acid and vortexed for 30 s. Then samples were placed into a preconditioned solid phase extraction cartridge (Phenomenex, 10 mg/mL, California, United States) and washed with 0.1% formic acid and 15% ethyl alcohol. After elution with methanol, lipid extract was evaporated and stored at -80 ℃ prior to lipidomic analysis.

#### LC-MS-based lipid profiling

The lipid extract was reconstituted in 100 μL methanol and vortexed for 5 min. After centrifuged (12,000 × g, 6 min, 4 °C), 80 μL of supernatant was transferred to a vial insert for analysis. AA-targeted lipid profiling was performed with a modified LC-MS methodology (LCMS-8050, Shimadzu, Kyoto, Japan) as previously reported [28]. LC-MS data were acquired using multiple-reaction monitoring mode with continuous ionization polarity switching. Chromatographic separation was achieved using a Phenomenex Kinetex C8 column (150 × 2.1 mm inner diameter, 2.6 μm film thickness). Mobile phase solutions were 0.1% formic acid for A and acetonitrile for B. The flow rate was maintained at 0.4 mL/min with the column temperature set to 40 °C. The solvent gradient was applied as follows: starting at 10% B and increasing to 25% B over 5 min, then to 35% B over 5 min, to 75% B over 10 min, to 95% B over 0.1 min, then held at 95% B for 4.9 min and returned to 10% B over 0.1 min. The injection volume was 5 µL for each run. The ionization was done in both positive and negative mode. Mass spectrometer running conditions were as follows: drying gas flow rate 15 L/min, heating gas flow rate 10 L/min, nebulizing gas flow rate 3 L/min, collision gas (argon gas, purity > 99.9995%) voltage 270 kPa, heat block temperature 400 °C, ESI interface temperature 300 °C, desolvation line temperature 250 °C.

#### Data processing and multivariate analyses

LC/MS raw data were assessed with LabSolutions (version 5.86, Shimadzu, Kyoto, Japan) for lipid identification, peak extraction, peak alignment, noise filtering and data extraction. Lipid response was calculated as the peak area ratio of the target analyte to the respective internal standard [29]. Then data were log-transformed for multivariate statistical analysis by SIMCA-P 14.1 (Umeta, Umeå, Sweden) and back-transformed for presentation as median (interquartile range) [29]. After auto-scaling, data were subjected to principal component analysis (PCA) to visualize the differences of lipid profile between DAPT and PQS+DAPT group. Orthogonal projection to latent structure-discriminant analysis (OPLS-DA) was further used to validate the lipidomic alterations by adding PQS to DAPT. The robustness of OPLS-DA model was validated by permutation testing. Lipid biomarkers that significantly differentiated between DAPT and PQS+DAPT group were determined based on the combinational thresholds of variable importance in projection (VIP) values larger than 1.0 obtained from OPLS-DA model and *P* values less than 0.05 obtained from Mann-Whitney *U* test.

### Statistical analyses

Statistics except of lipidomic analysis were performed using SPSS (version 21.0, Chicago, Illinois, USA). Normally distributed variables were presented as mean ± SEM. For multiple group comparisons, one-way ANOVA was performed with LSD as post hoc correction. Differences were considered statistically significant at *P* < 0.05.

## Results

### PQS+DAPT enhanced inhibitory effect on platelet aggregation

**Fig. 1:**
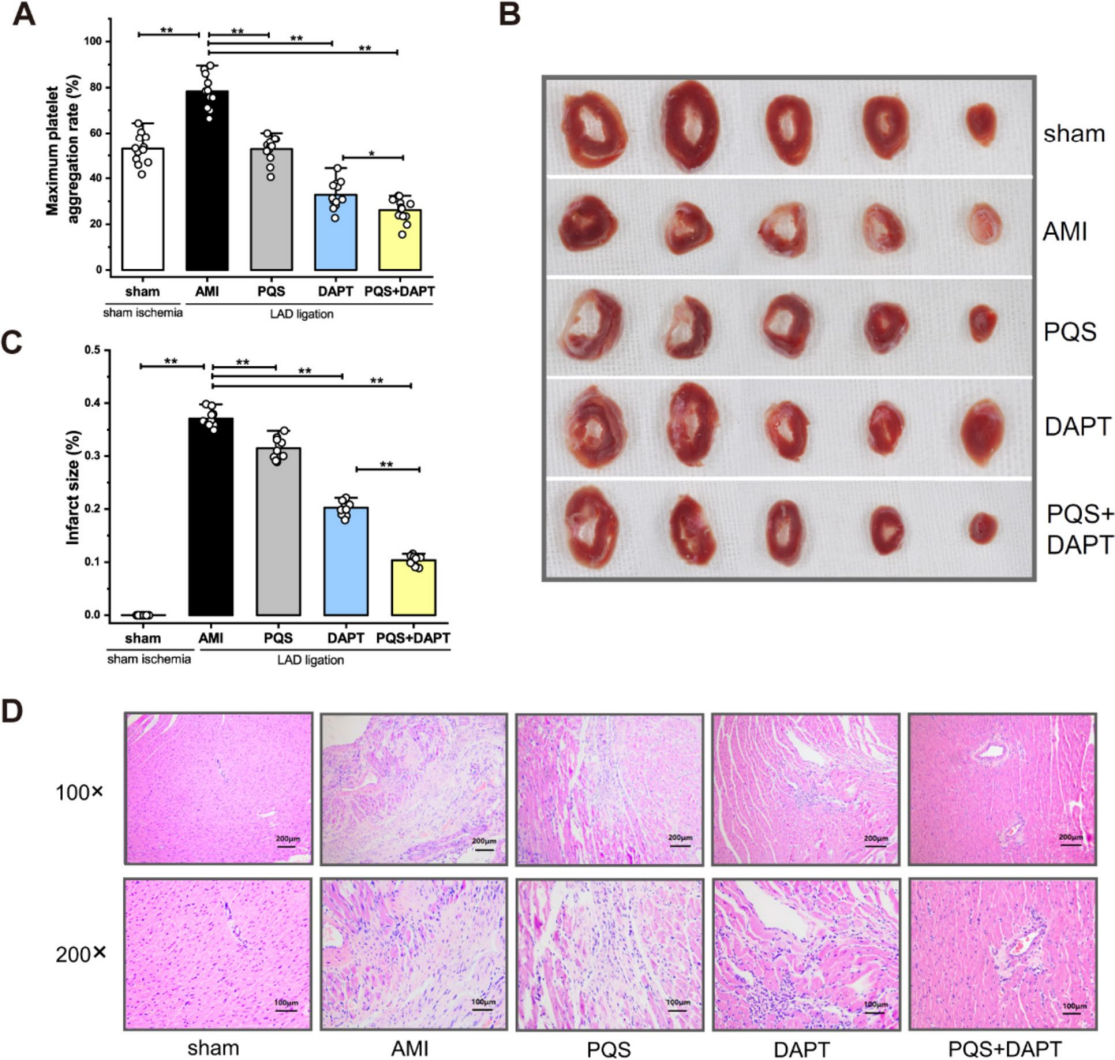
PQS+DAPT further decreased platelet aggregation, attenuated MI expansion and cardiac injury in AMI rats. **A** ADP-induced MPAR measured by light transmission aggregometry revealed potentiated inhibition of platelet aggregation by PQS+DAPT compared with DAPT. Each dot represents one rat (*n* = 12). **B** MI expansion was visualized by TTC staining and infarct size was measured using Image J software. PQS+DAPT further diminished infarct size relative to DAPT. Each dot represents one rat (*n* = 10). **C** Representative images of TTC staining. **D** Representative images of myocardial histopathology by H&E staining (× 100, Scale bar: 200 μm; × 200, Scale bar: 100 μm). Multiple group comparison was conducted by one-way ANOVA. *P < 0.05, **P < 0.01. Data were mean ± SEM in all panels

Platelet activation and thrombus formation is a multistage process including shape change, platelet adhesion and aggregation [30]. We previously revealed more potent inhibition of platelet adhesion to oxidized low-density lipoprotein injured HUVEC by PAS+DAPT compared with DAPT [12]. The present study further studied the anti-aggregatory effect of the joint therapy in a rat model of AMI. Platelet aggregation was analyzed in citrate-anticoagulated PRP to avoid agonists released from erythrocytes [31]. At 29 days after surgery, maximum platelet aggregation rate (MPAR) induced by ADP increased markedly in AMI rats compared with those with sham operation, indicating enhanced platelet reactivity post MI (*P* < 0.01, Fig. 1A). Rat treated with PQS alone and in combination with DAPT showed significantly decreased MPAR compared with those in AMI group (both *P* < 0.01, Fig. 1A). Furthermore, PQS+DAPT decreased MPAR compared with DAPT (*P* < 0.05, Fig. 1A), suggesting a reinforced anti-aggregatory effect of the combined therapy.

### PQS+DAT ameliorated MI expansion

Irregulated platelet activation may induce thrombotic occlusion of vessel lumen, leading to myocardial infarction expansion [32]. While platelet adhesion represents the initial phase of thrombosis, platelet aggregation is the key step of platelet accumulation amplifying thrombus formation [13]. We next addressed the impact of PQS, DAPT or the combined therapy on infarct size. Rats subjected to permanent occlusion of LAD coronary artery exhibited transmural infarction, as shown in Fig. 1B. PQS monotherapy, DAPT and PQS+DAPT suppressed MI expansion post MI (both *P* < 0.01 vs AMI rats, Fig. 1C), with a significantly reduced infarct size in response to PQS+DAPT compared with DAPT (*P* < 0.01, Fig. 1C). Thus, these findings indicated that adding PQS to DAPT achieved greater platelet inhibition and further ameliorated MI progression.

### PQS+DAPT alleviated cardiac injury

To determine the influence of PQS, DAPT or the combined therapy on myocardial histopathology, H&E staining was performed (Fig. 1D). Rats subjected to sham surgery exhibited normal myocardial structural patterns. Hearts of AMI rats exhibited significant morphologic changes at 30 days post-MI, showing massive dense collagenous scar with a few vital myocytes. In PQS-treated rats, areas of cardiac damage were slightly diminished, characterized with narrowed collagen deposition and increased cellularity. DAPT significantly reduced the extent of myocardial necrosis with limited scar formation. Intriguingly, adding PQS to DAPT further lessen the size of infarct and markedly alleviated the degree of cardiac injury.

### PQS mitigated DAPT-induced gastric mucosal injury

**Fig. 2:**
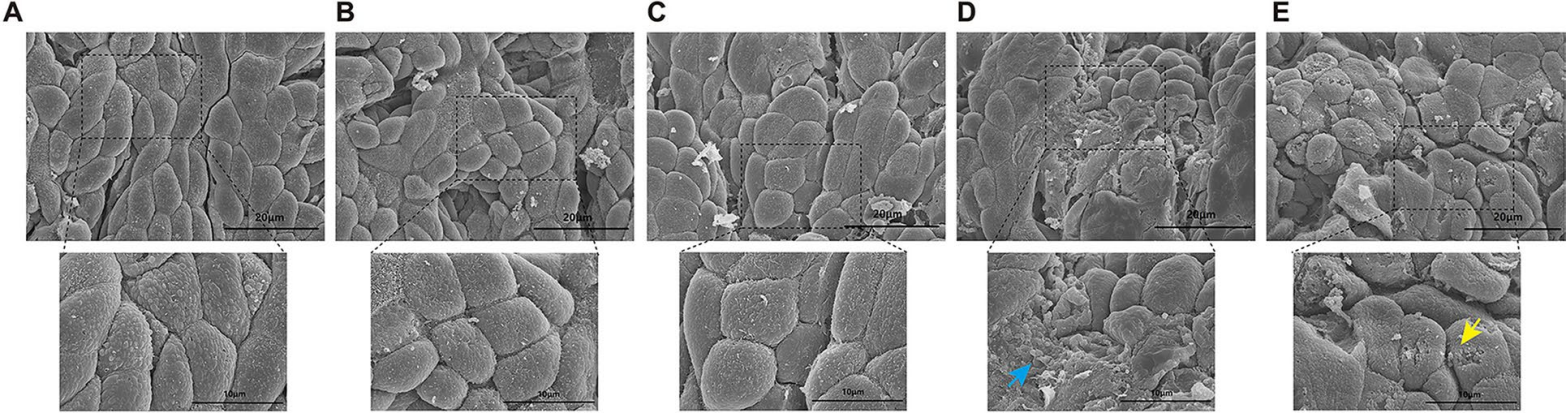
PQS mitigated DAPT-induced gastric mucosal injury. Representative images of gastric antrum mucosa by SEM in **A** sham group, **B** AMI group, **C** PQS group, **D** DAPT group and **E** PQS+DAPT group. (× 5000, Scale bar: 10 μm; insert image × 2000, Scale bar: 20 μm). DAPT caused a vast ablation of the gastric surface epithelium (shown in blue arrow). In PQS+DAPT treated rats, mucosa injury was attenuated, as demonstrated by a few apical erosions (shown in yellow arrows)

It has been reported that ultrastructural damage to the gastric epithelium occurs during short-term administration of ASA with no area of the stomach resistant to the injury. The most frequently and severely affected site is the gastric antrum [33]. In the present study, antrum specimen was collected for better observation of the gastric injury. Rats treated with saline and PQS exhibited normal cobblestone structure with regular arrangement of epithelial cells (Fig. 2A-C). After DAPT administration, overt epithelium deformation was observed. Moreover, DAPT caused a vast ablation of the surface epithelium; thereby, the honeycomb structure of the denuded lamina propria became apparent (Fig. 2D). By contrast, gastric mucosa in PQS+DAPT-treated rats showed a few apical erosions while the severity of surface desquamation was diminished (Fig. 2E), indicating alleviated gastric injury by adding PQS to DAPT.

### AA-targeted lipidomic analysis revealed alteration of eicosanoids metabolism in plasma by adding PQS to DAPT

**Fig. 3:**
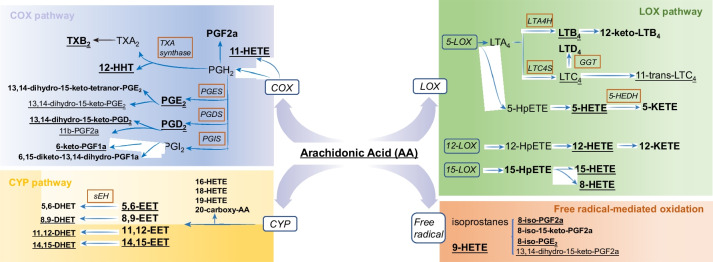
Eicosanoid biosynthesis from AA via COX, LOX, CYP enzymatic pathways as well as by free radical-mediated oxidation. COX, LOX, CYP are shown in blue ovals and downstream enzymes are shown in red boxes. Note that eicosanoid detected in plasma are in bold and those detected in mucosa are underlined

**Fig. 4:**
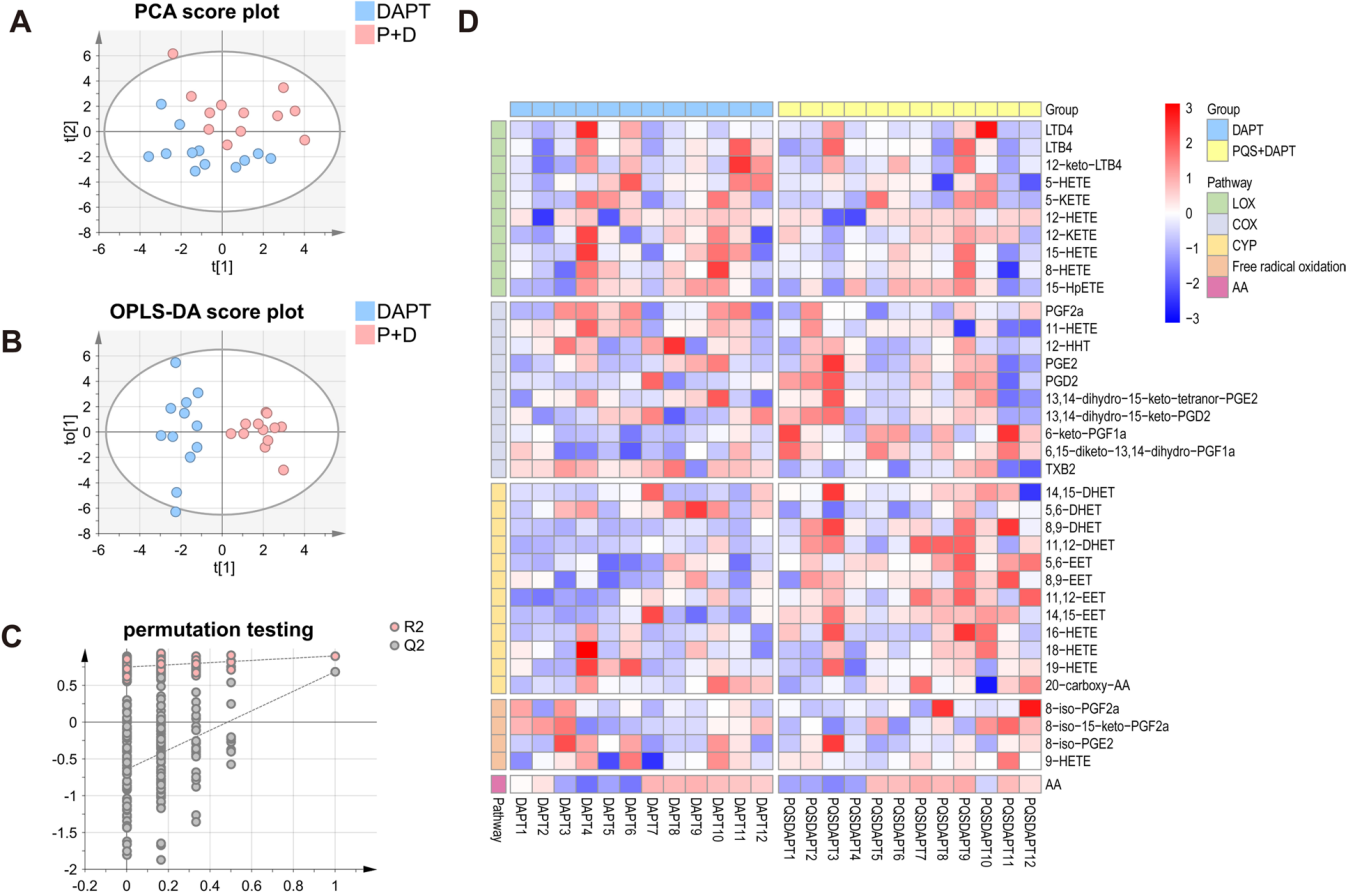
Lipidomic analysis for plasma unraveled significantly altered eicosanoids profile by adding PQS to DAPT. Multivariate analysis results shown in **A** PCA score plot and **B** OPLS-DA score plot demonstrated distinct discrimination of plasma lipid profile in DAPT group compared with PQS+DAPT group. **C** Validation of OPLS-DA model by permutation testing. **D** Heatmap analysis provided a metabolic snapshot of the plasma lipid profile in the two groups. P+D, PQS+DAPT

AA-derived eicosanoids produced by platelets and endothelial cells exert expansive role on platelet biology that significantly regulate platelet reactivity [16]. They can be secreted by cells and stable metabolites may accumulate in circulation. To determine the impact of PQS+DAPT, relative to DAPT, on eicosanoids profile in plasma, a robust LC-MS-based, AA-targeted lipidomic profiling was performed after 30 days of drug intervention. The platform determined 87 AA-derived lipids including eicosanoids and breakdown products, among which 37 were detectable in plasma (Fig. 3 and Table S1). The preprocessed LC/MS data were subjected to PCA analysis and samples with similar lipid profiles were clustered closely in the score plot of PCA. As shown in Fig. 4A, distinct clusters for plasma lipid profile in DAPT and PQS+DAPT group was observed, suggesting alteration of eicosanoids metabolism by adding PQS to DAPT. Supervised OPLS-DA analysis further confirmed the metabolic differences between DAPT and PQS+DAPT groups (Fig. 4B). The robustness of PLS-DA model was validated by 200-repeated permutation test (Fig. 4C). The heatmap provided a metabolic snapshot of the plasma lipid profile (Fig. 4D). Thirteen lipids with VIP value higher than 1.0 were considered potential pharmacological biomarkers in response to PQS+DAPT compared with DAPT (Table S2).

**Fig. 5:**
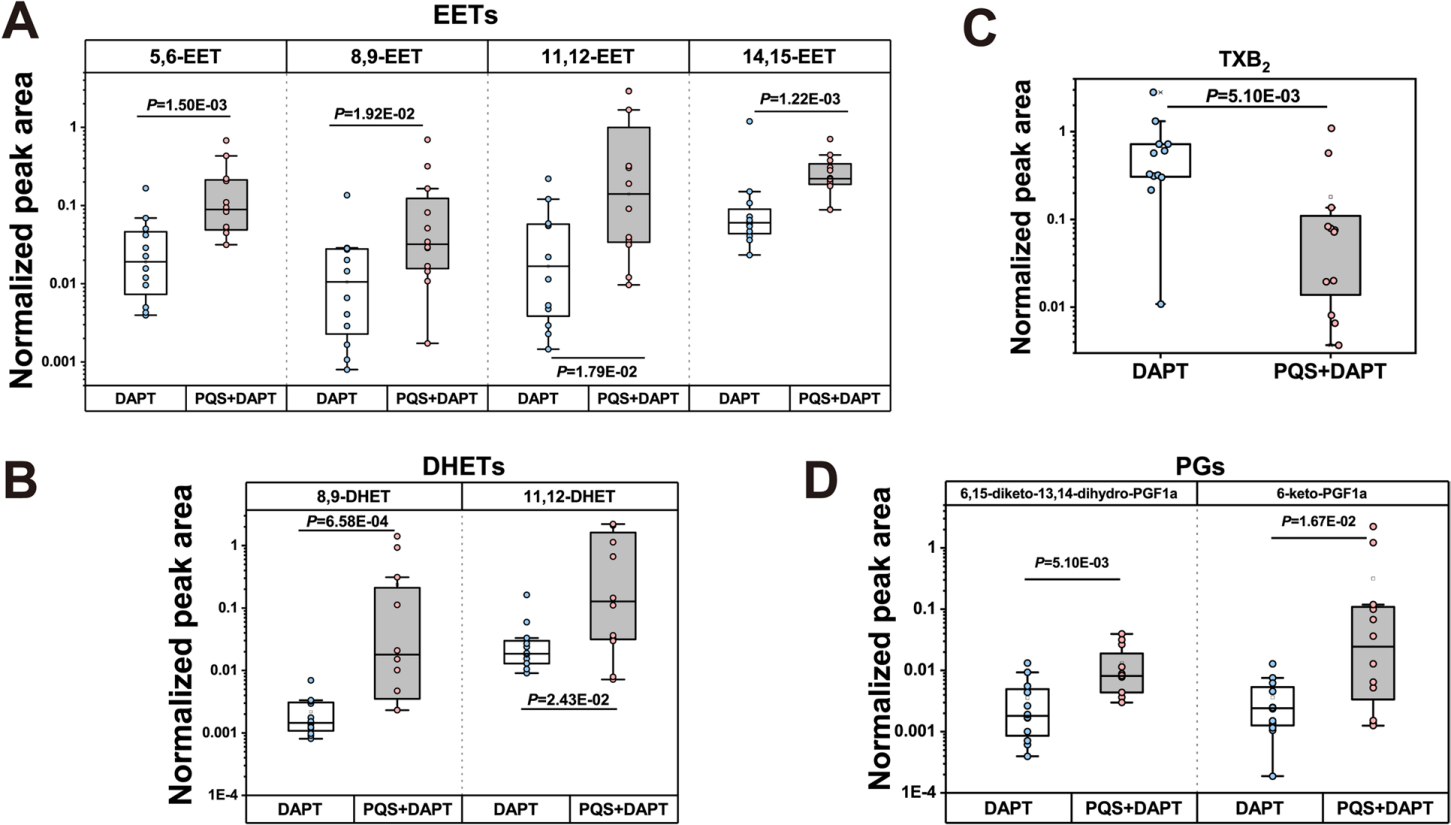
Lipidomic analysis unraveled significantly altered plasma eicosanoids by adding PQS to DAPT. Plasma level of **A** EETs, **B** DHETs, **C** TXB_2_ and **D** PGI_2_ downstream lipids were shown in box plots. Each dot represents one rat (*n* = 12). Data were presented as median (interquartile range) in all panels

To figure out lipids that were significantly up-/ down-regulated by PQS+DAPT compared with DAPT, Mann-Whitney *U* test was used. 9 out of 13 lipids remained significantly differentiated between the two groups (Fig. 5A-D, Table S3, and receiver operating characteristic analysis for these 9 lipids between DAPT group and PQS+DAPT group were shown in Fig. S2A-D). Specifically, there were significant increases in the abundance of epoxyeicosatrienoic acids (EETs) (5,6-EET, 8,9-EET, 11,12-EET, 14-15-EET) and downstream dihydroxyeicosatrienoic acids (DHETs) (8,9-DHET, 11-12DHET), all from CYP pathway, in PQS+DAPT group as compared to DAPT group (Fig. 5A-B). Moreover, PQS supplementation to DAPT resulted in a roughly 6-fold decrease in plasma level of thromboxane (TX)B_2_, while let to significantly up-regulation of 6-keto-PGF1a and 6,15-diketo-13,14-dihydro-PGF1a, downstream metabolites of PGI_2_ (Fig. 5C-D).

### AA-targeted lipidomic analysis uncovered alteration of eicosanoids metabolism in gastric mucosa by adding PQS to DAPT

**Fig. 6:**
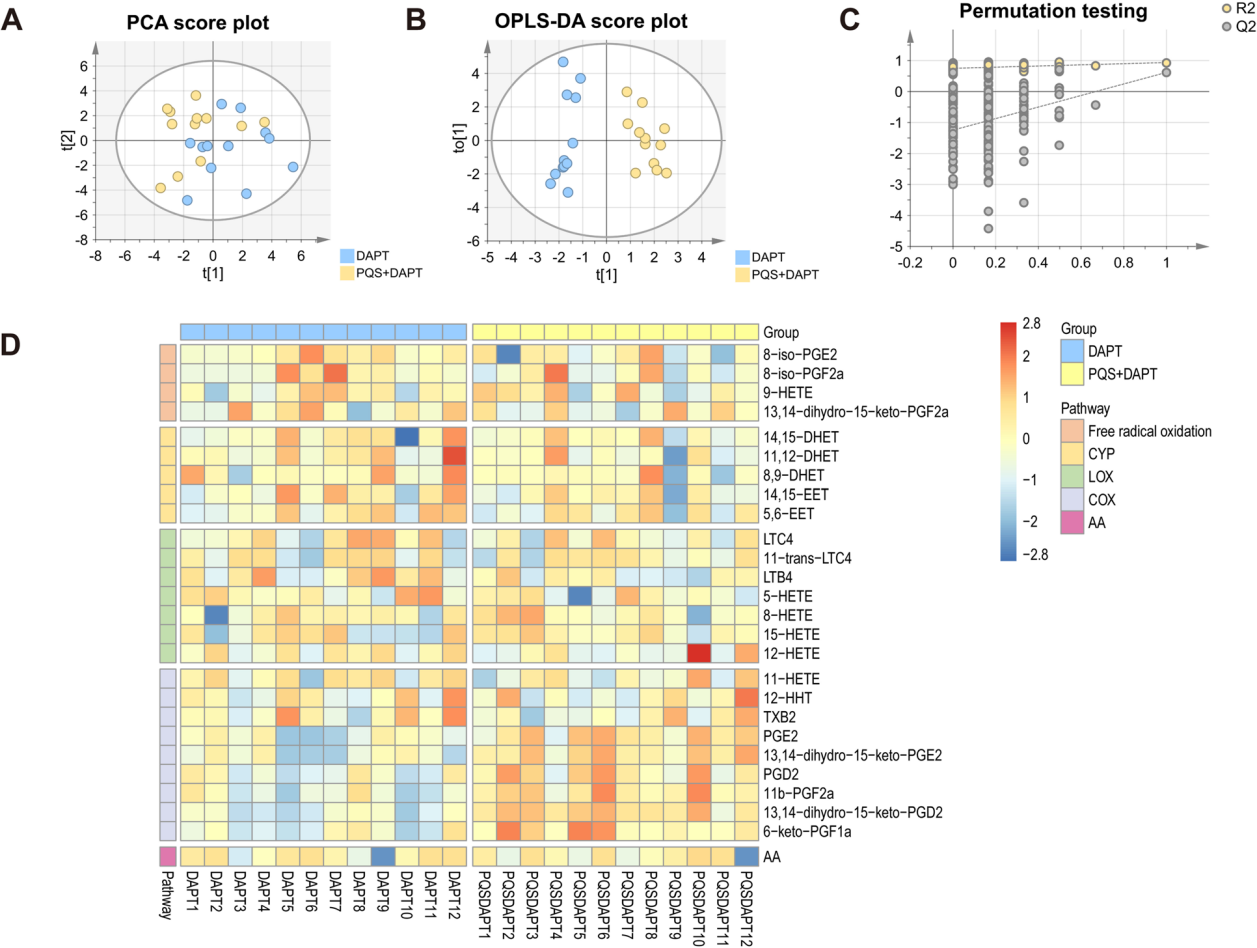
Lipidomic analysis for gastric mucosa uncovered significantly altered eicosanoids profile by adding PQS to DAPT. Multivariate analysis results shown in **A** PCA score plot and **B** OPLS-DA score plot demonstrated distinct discrimination of gastric lipid profile in DAPT group compared with PQS+DAPT group. **C** Validation of OPLS-DA model by permutation testing. **D** Heatmap analysis visualized the gastric lipid profile in the two groups

AA-derived eicosanoids in gastric lumen are locally acting bioactive lipids that play homeostatic roles ranging from regulating vascular leakage, barrier formation to protecting mucosal integrity [34]. We harvested gastric mucosa from antrum, the most vulnerable site to ASA-related GI toxicity, to investigate mucosal lipidomic alteration in response to PQS+DAPT, relative to DAPT. A total of 26 lipids were identified in antrum mucosa by LC/MS analysis as shown in Fig. 3 and Table S4. Score plot of PCA analysis revealed separated clusters for gastric lipid profile in DAPT and PQS+DAPT group with partly overlapping 95% confidence intervals, indicating that gastric lipidomic profile was altered by adding PQS to DAPT (Fig. 6A). Next, OPLS-DA analysis was conducted and validated as shown in Fig. 6B-C. Score plot of OPLS-DA model demonstrated more clear classification of lipid profile in the two groups. Heatmap analysis visualized the gastric lipid profile in both groups, suggesting up-regulated synthesis of lipids downstream of AA/COX pathway in response to PQS+DAPT, relative to DAPT (Fig. 6D). As Expected, 7 lipids with VIP higher than 1.0 were all derived from AA/COX pathway (Table S5).

**Fig. 7:**
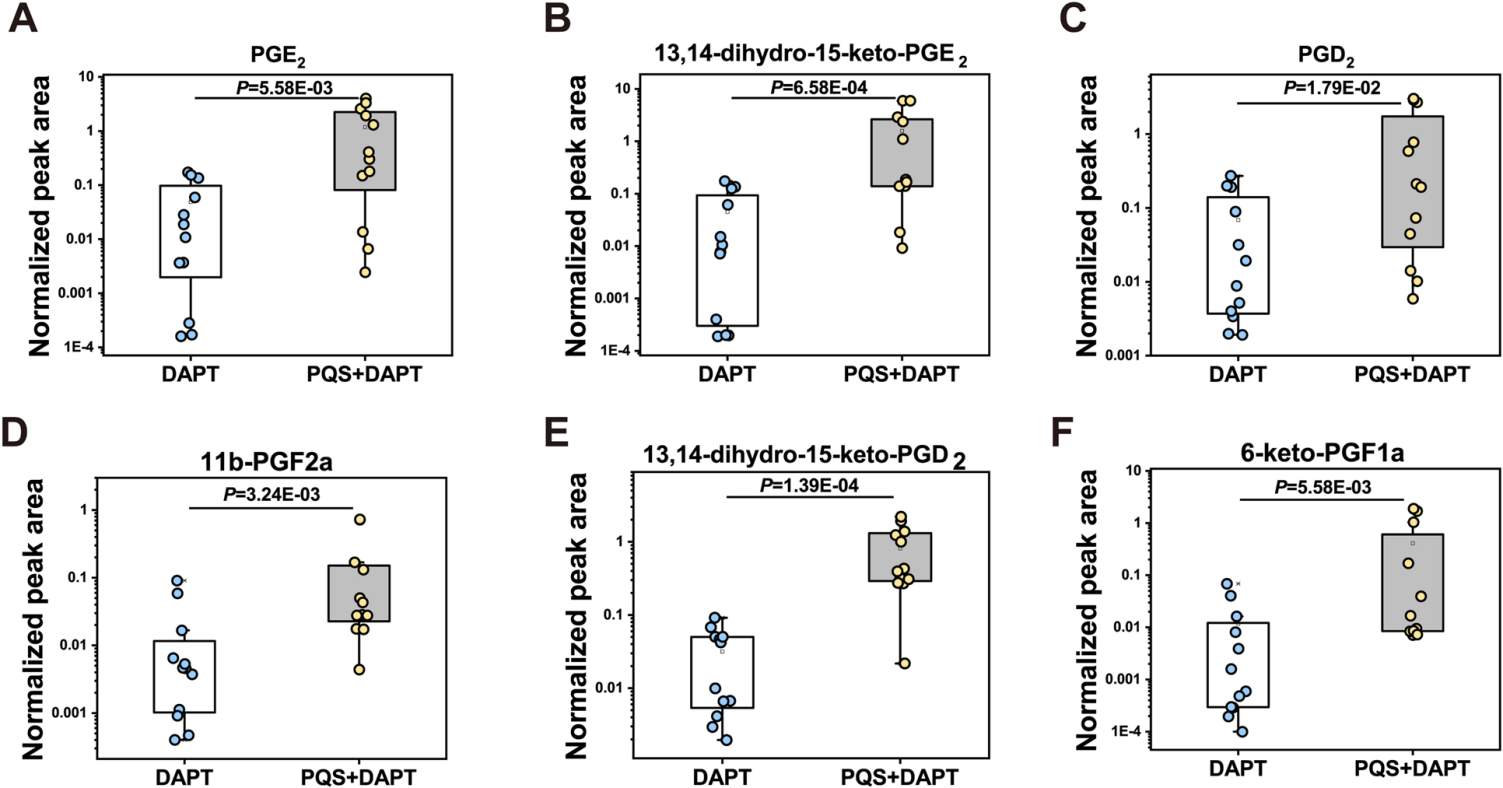
Lipidomic analysis unraveled significantly altered gastric eicosanoids by adding PQS to DAPT. Plasma level of **A** PGE_2_, **B** 13,14-dihydro-15-keto-PGE_2_, **C** PGD_2_, **D** 11b-PGF2a, **E** 13,14-dihydro-15-keto-PGD_2_ and **F** 6-keto-PGF1a were shown in box plots. Each dot represents one rat (*n* = 12). Data were presented as median (interquartile range) in all panels

Six out of 7 lipids reached statistical significance by Mann-Whitney *U* test, reflecting the eicosanoids significantly changed by PQS+DAPT treatment compared with DAPT (Fig. 7A-F, Table S6, and receiver operating characteristic analysis for these 6 lipids between DAPT group and PQS+DAPT group were shown in Fig. S2E–G). Adding PQS to DAPT resulted in approximately 20-fold increase of gastric PGE_2_ and its breakdown product 13,14-dihydro-15-keto-PGE_2_ relative to DAPT (Fig. 7A-B). Similarly, we observed over tenfold elevation of gastric PGD_2_ and 11b-PGF2a, 13,14-dihydro-15-keto-PGD_2_, 6-keto-PGF1a (downstream metabolites of PGD_2_ and PGI_2_) in PQS+DAPT group compared with DAPT group (Fig. 7C-F).

## Discussion

**Fig. 8:**
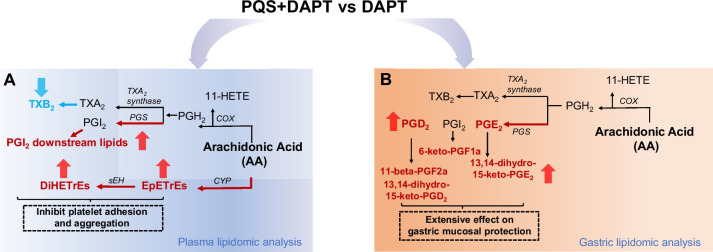
Mechanisms underlying the enhanced platelet inhibition and gastroprotection by PQS+DAPT was associated with eicosanoids metabolic modulation. Regulated pathway in **A** plasma and **B** gastric mucosa. The arrows indicated upregulated (red arrows) or downregulated (blue arrows) in PQS+DAPT group relative to DAPT group. Enzymes are illustrated in italics. PGS indicates PG synthase; sEH, soluble epoxide hydrolase

This study demonstrated that adding PQS to DAPT achieved more potent antiplatelet effect with ameliorated MI expansion and cardiac injury in a rat model of AMI. PQS, as an adjunct to DAPT, mitigated DAPT-related gastric damage illustrated by SEM. By lipidomic approaches, we comprehensively characterized metabolic alterations of AA-derived eicosanoids in response to PQS+DAPT, relative to DAPT, in rat plasma and gastric mucosa. Plasma lipidomic analysis revealed markedly increased EETs, PGI_2_, potent inhibitors of platelet adhesion and aggregation, and significantly decreased TXB_2_, stable form of a potent platelet agonist-TXA_2_, by adding PQS to DAPT, suggesting the improved platelet inhibition of PQS+DAPT may be partly attributed to up-regulated AA/EET, AA/PGI_2_ and down-regulated AA/TXA_2_ metabolism. Additionally, PQS supplementation to DAPT markedly elevated gastric PGE_2_, PGD_2_ and their downstream lipids, which play pivotal roles in mucosal defense. The gastroprotective action of PQS on DAPT-induced gastric injury could be associated with locally augmented PG production in gastric mucosa (Fig. 8).

Suboptimal platelet inhibition despite the use of DAPT has been reported commonly in the setting of ACS [35, 36]. Here, we found a synergistic antiaggregatory effect when adding PQS to DAPT in AMI rats. The benefits of PQS have been generally ascribed to ginsenosides [10], including mainly ginsenoside Rb1, Rb2, Rc, Rd, Re and Rg1. Our finding is in accordance with an earlier in vitro study showing that ginsenoside Rg1 potently inhibited platelet aggregation [37]. Notably, under ischemic stress post MI, uncontrolled platelet activation and aggregation would induce acute vessel occlusion, leading to ongoing MI expansion [38]. Presently, adding PQS to DAPT resulted in significantly reduced MI expansion and alleviated cardiac injury, which was associated with potentiated platelet inhibition. Moreover, inadequate platelet inhibition despite DAPT has been associated with 2-9 fold increased risk of major cardiovascular events [36]. We recently demonstrated that PQS+DAPT further reduced the incidence of primary composite endpoint (cardiac death, nonfatal MI and urgent revascularization) in patients underwent percutaneous coronary intervention [39]. Therefore, these findings suggested that PQS provided incremental benefit in platelet inhibition when added to DAPT, which might contribute to cardiac protection under ischemic conditions.

AA-derived eicosanoids are essential for maintaining vascular homeostasis through actions on platelets and endothelial cells [16]. Acting as autocrine or paracrine mediators, eicosanoids can be secreted by tissues and stable metabolites will accumulate in plasma [40]. Eicosanoids metabolic patterns are highly sensitive to physiological stimuli including drug interventions [41]. Currently, plasma lipidomic profile provided an accurate snapshot of metabolic alterations after adding PQS to DAPT. We observed significantly increased abundance of CYP-derived EETs and their breakdown metabolites (8,9-DHET, 11-12DHET) resulted from PQS+DAPT compared with DAPT. EETs are potent vasodilators primary generated by endothelial cells [42]. Besides, EETs exert anti-inflammatory effects in blood vessels, limiting platelet aggregation [43], which may account for the greater anti-aggregatory effect of PQS+DAPT in the present study. In addition, EETs were shown to cause membrane hyperpolarization of platelets that ultimately suppress platelet adhesion to endothelial cells [44]. Collectively, PQS potentiated DAPT-mediated inhibition of platelet adhesion, as reported previously [12], and aggregation, effects that may partially ascribed to upregulation of AA/EET metabolism.

Furthermore, the addition of PQS to DAPT augmented synthesis of PGI_2_ while decreased production of TXA_2_, as evidenced by a roughly 10-fold increase in plasma 6-keto-PGF1a, 3-fold increase in plasma 6,15-diketo-13,14-dihydro-PGF1a (PGI_2_ downstream metabolites) and a 4-fold decrease in plasma TXB_2_ (stable form of TXA_2_). This is consistent with our previous finding that adding PQS to DAPT markedly increased 6-keto-PGF1α/TXB_2_ ratio in rat plasma [14]. The primary antiplatelet effect of ASA is to acetylate platelet COX-1 and thereby inhibit the synthesis of TXA_2_, a powerful agonist for platelet activation and aggregation [45]. However, insufficient inhibition of TXA_2_ biosynthesis despite ASA has been associated with an increased risk of cardiovascular events [46]. The downregulated TXA_2_ metabolism in response to PQS+DAPT may underlie the enhanced anti-aggregatory effect of the integrated therapy, which could also contribute, at least in part, to the reduced cardiovascular events as recently reported [39]. In contrast, PGI_2_ counteract the prothrombotic properties of TXA_2_ and efficiently inhibits platelet activation [19]. Interestingly, EETs were shown to suppress TXB_2_ formation in vivo [47]. Taken together, adding PQS to DAPT reinforced platelet inhibition by maintaining antithrombotic miliue via promoting AA/PGI_2_ and AA/EET synthesis while suppressing AA/TXA_2_ metabolism.

ASA, even at low doses (75–225 mg/day), is known to cause various GI damage, ranging from mucosal injury to more sever GI bleeding, the latter is independently associated with mortality [8]. Of note, it has been reported that more than 60% of patients taking low dose ASA show endoscopic gastric erosions [48], especially in the gastric antrum [24]. Indeed, ASA-induced gastric injury is substantially more common than overt bleeding and has been considered as a surrogate for the propensity of GI hemorrhagic complications. The addition of CLP to ASA further increases the risk of gastric injury by impairing mucosal healing [49]. Nearly 90% of DAPT users develop endoscopic gastric mucosal injury [50]. In the present study, gastric antrum tissue was selected for better observation of gastric injury. DAPT causes significant mucosal damage characterized by a vast ablation of epithelium and the exposure of the lamina propria, which was attenuated by PQS administration. Unfortunately, DAPT-related gastric injury is largely asymptomatic [51], rendering suboptimal utilization of gastroprotective regimen. Although guidelines recommend concomitant use of proton pump inhibitors (PPIs) in patients receiving DAPT to reduce GI bleeding [52], PPI has been found to mitigate the anti-aggregatory effect of ASA [53]. In light of the above-mentioned evidence, the pleiotropic effect of PQS on platelet inhibition and gastroprotection suggest it as a potential supplementary drug to improve the benefit of DAPT.

Eicosanoids play critical roles in gastric physiology [17]. In GI tract, they mainly function in site of their production, so mucosal tissue was harvested for lipidomic analysis. Presently, we found consistent increase of COX-derived PGs (PGE_2_, PGD_2_, their downstream metabolites and stable form of PGI_2_) in gastric mucosa after adding PQS to DAPT. Notably, PQS supplementation elevated gastric PGE_2_ levels more than 20-fold relative to DAPT. ASA-induced gastric injury is primarily due to inhibition of COX activity, resulting in reduced synthesis of mucosal PGs [54]. PGs are crucial mediators for gastroprotection. Almost all of the mucosal defense mechanisms are stimulated or facilitated by PGs, including stimulating mucus secretion, increasing blood flow, accelerating epithelial restitution and mucosal healing [55]. Specifically, PGE_2_ administration was reported to attenuate gastric mucosal damage induced by DAPT in rats (unpublished result). Therefore, the gastroprotective effect of PQS on DAPT-induced gastric injury could be partially attributed to the elevated PGs synthesis in gastric mucosa.

The present study evidenced synergistic antiplatelet effect and gastroprotective action by combining PQS with DAPT in AMI rats, which were associated with regulation of eicosanoids metabolism. Some limitations remain. Ginsenosides, the bioactive constituents of PQS, belong to terpenoids class. Phytochemicals in herbal medicine, including terpenoids, are found to broadly change the metabolic patterns of oxylipins by influencing enzymes activity [56], which may explain the lipidomic alterations in current study. Cellular targets and molecular mechanisms underlying the joint effect remain to be elucidated. On the other hand, as platelets are crucial in thrombosis and hemostasis, whether adding PQS to DAPT prolongs bleeding time will be explored in the future.

## Conclusions

In summary, cotreatment with PQS and DAPT achieved better antiplatelet effect attributed to up-regulating AA/PGI_2_, AA/EET synthesis and down-regulating AA/TXA_2_ metabolism. PQS mitigated DAPT-induced gastric mucosa injury via promoting mucosal PG production. PQS may provide incremental benefits as a complementary agent to DAPT in the treatment of ACS.

### Supplementary Information

**Additional file 1. **Supplementary materials.

## Data Availability

The datasets used and/or analysed during the current study are available from the corresponding author on reasonable request.

## Abbreviations

DAPT: Dual antiplatelet therapy
ASA: Aspirin
CLP: Clopidogrel
ACS: Acute coronary syndromes
MI: Myocardial infarction
GI: Gastrointestinal
PQ: *Panax quinquefolius*
PQS: *Panax quinquefolius* Saponins
HUVEC: Human umbilical vein endothelial cell
AA: Arachidonic acid
COX: Cyclooxygenase
LOX: Lipoxygenase
CYP: Cytochrome P450
PG: Prostaglandin
ADP: Adenosine diphosphate
TTC: 2,3,5-Triphenyltetrazolium chloride
AMI: Acute myocardial infarction
TX: Thromboxane
LT: Leukotriene
HETE: Hydroxyeicosatetraenoic acid
LC-MS: Liquid chromatography-mass spectrometer
LAD: Left anterior descending
H&E: Hematoxylin and eosin
PRP: Platelet-rich plasma
PPP: Platelet-poor plasma
LV: Left ventricle
SEM: Scanning electron microscope
PCA: Principal component analysis
OPLS-DA: Orthogonal projection to latent structure-discriminant analysis
VIP: Variable importance in projection
MPAR: Maximal platelet aggregation rate
EET: Epoxyeicosatrienoic acid
DHET: Dihydroxyeicosatrienoic acid
TX: Thromboxane
PPI: Proton pump inhibitor
