# Supplementary material for: Panax quinquefolius saponins combined with dual antiplatelet therapy enhanced platelet inhibition with alleviated gastric injury via regulating eicosanoids metabolism
Source: BMC Complement Med Ther. 2023 Aug 18;23:289. doi: 10.1186/s12906-023-04112-7 (PMC10436642; doi:10.1186/s12906-023-04112-7)
Supplement: Supplementary file 1 — Additional file 1. Supplementary materials. [file 12906_2023_4112_MOESM1_ESM.docx]

**Supplementary material**

*Panax quinquefolius* saponins combined with dual antiplatelet therapy enhanced platelet inhibition with alleviated gastric injury via regulating eicosanoids metabolism

**Contents**

1. **Fig. S1.** Chemical structure of major bioactive constituents of PQS.
2. **Table S1** AA-derived eicosanoids detected in rat plasma.
3. **Table S2** The information of 13 plasma eicosanoids with VIP higher than 1.
4. **Table S3** 9 lipids that were significantly up-/ down-regulated by PQS+DAPT compared with DAPT in rat plasma.
5. **Fig. S2.** Receiver operating characteristic analysis for significantly differential lipids between DAPT group and PQS+DAPT group in plasma and gastric mucosa.
6. **Table S4** AA-derived eicosanoids detected in rat gastric mucosa.
7. **Table S5** The information of 7 plasma eicosanoids with VIP higher than 1.
8. **Table S6** 6 lipids that were significantly up-/ down-regulated by PQS+DAPT compared with DAPT in rat gastric mucosa.

**Fig. S1.** Chemical structure of major bioactive constituents of PQS. (A) ginsenoside Rb1, (B) ginsenoside Rb2, (C) ginsenoside Rc, (D) ginsenoside Rd, (E) ginsenoside Re and (F) ginsenoside Rg1.


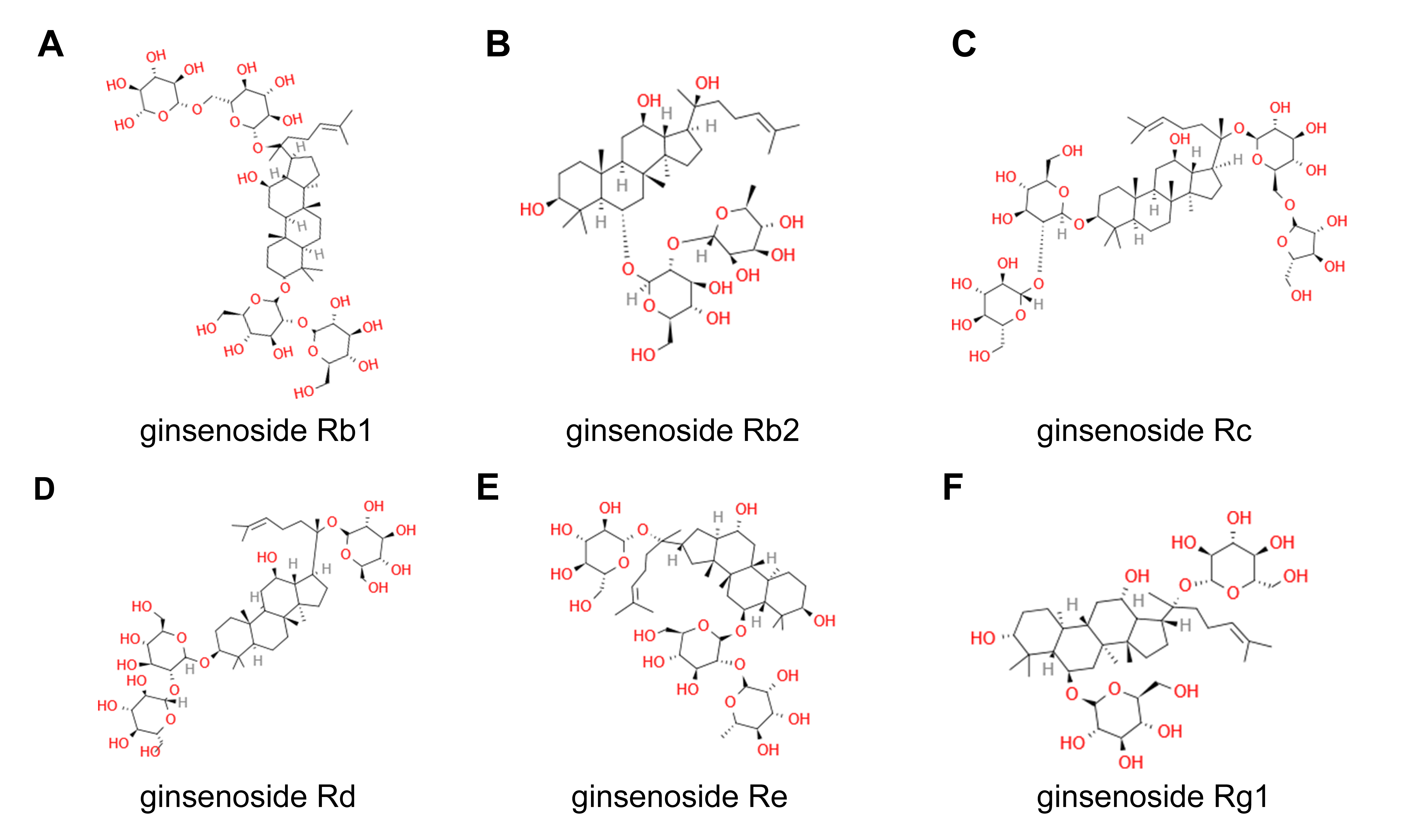


**Table** **S1** AA-derived eicosanoids detected in rat plasma. Significantly altered eicosanoids between DAPT group and PQS+DAPT group were shown in bold.

| No. | Eicosanoid name | Enzyme | Human Metabolome Database ID | KEGG compound ID |
| --- | --- | --- | --- | --- |
| 1 | **6-keto-PGF1a** | COX | HMDB0002886 | C05961 |
| 2 | 13,14-dihydro-15-keto-tetranor-PGE_2_ | COX | - | - |
| 3 | **6,15-diketo-13,14-dihydro-PGF1a** | COX | HMDB0001979 | - |
| 4 | 8-iso-PGF2a | free radical-catalyzed | HMDB0005083 | C13809 |
| 5 | **TXB_2_** | COX | HMDB0003252 | C05963 |
| 6 | 8-iso-15-keto-PGF2a | free radical-catalyzed | HMDB0005077 | - |
| 7 | PGF2a | COX | HMDB0001139 | C00639 |
| 8 | 8-iso-PGE_2_ | free radical-catalyzed | HMDB0005844 | - |
| 9 | PGE_2_ | COX | HMDB0001220 | C00584 |
| 10 | PGD_2_ | COX | HMDB0001403 | C00696 |
| 11 | LTD_4_ | 5-LOX | HMDB0003080 | C05951 |
| 12 | 13,14-dihydro-15-keto-PGD_2_ | COX | HMDB0060042 | - |
| 13 | LTB_4_ | 5-LOX | HMDB0001085 | C02165 |
| 14 | 12-keto-LTB_4_ | 5-LOX | HMDB0004234 | C05949 |
| 15 | 5,6-DHET | CYP | HMDB0002343 | C14772 |
| 16 | **14,15-DHET** | CYP | HMDB0002265 | C14775 |
| 17 | 12-HHT | COX | HMDB0012535 | C20388 |
| 18 | 11,12-DHET | CYP | HMDB0002314 | C14774 |
| 19 | **8,9-DHET** | CYP | HMDB0002311 | C14773 |
| 20 | 20-carboxy-AA | CYP | - | - |
| 21 | 19-HETE | CYP | HMDB0011136 | C14749 |
| 22 | 18-HETE | CYP | HMDB0006245 | - |
| 23 | 16-HETE | 12-LOX | HMDB0004680 | C14778 |
| 24 | 15-HETE | 15-LOX | HMDB0003876 | C04742 |
| 25 | 11-HETE | COX | HMDB0004682 | C14780 |
| 26 | 8-HETE | 15-LOX | HMDB0004679 | C14776 |
| 27 | 15-HpETE | 15-LOX | HMDB0062688 | - |
| 28 | 12-HETE | 12-LOX | HMDB0006111 | C14777 |
| 29 | 9-HETE | free radical-catalyzed | HMDB0010222 | - |
| 30 | 5-HETE | 5-LOX | HMDB0011134 | C04805 |
| 31 | 12-KETE | 12-LOX | HMDB0013633 | C14807 |
| 32 | **14,15-EET** | CYP | HMDB0002283 | - |
| 33 | **11,12-EET** | CYP | HMDB0004673 | - |
| 34 | 5-KETE | 5-LOX | HMDB0010217 | C14732 |
| 35 | **8,9-EET** | CYP | HMDB0002232 | C14769 |
| 36 | **5,6-EET** | CYP | HMDB0002190 | C14768 |
| 37 | AA | - | HMDB0001043 | C00219 |

**Table S2** The information of 13 plasma eicosanoids with VIP higher than 1.

| No. | Eicosanoid name | VIP rank | m/z |
| --- | --- | --- | --- |
| 1 | 6,15-diketo-13,14-dihydro-PGF1a | 1.71 | 113.1 |
| 2 | 8,9-DHET | 1.63 | 127.1 |
| 3 | 6-keto-PGF1a | 1.60 | 245.2 |
| 4 | TXB_2_ | 1.58 | 195.1 |
| 5 | 5,6-EET | 1.47 | 191.1 |
| 6 | 14,15-EET | 1.45 | 113.1 |
| 7 | 11,12-DHET | 1.29 | 167.1 |
| 8 | 11,12-EET | 1.27 | 167.1 |
| 9 | 8,9-EET | 1.26 | 127.1 |
| 10 | 5,6-DHET | 1.22 | 145.1 |
| 11 | 16-HETE | 1.18 | 233.2 |
| 12 | 11-HETE | 1.04 | 167.1 |
| 13 | 15-HETE | 1.01 | 219.2 |

Abbreviations: VIP, variable importance in projection; m/z, mass number/charge number.

**Table S3** 9 lipids that were significantly up-/ down-regulated by PQS+DAPT compared with DAPT in rat plasma.

| No. | Eicosanoid name | FC | *P* value | AUC |
| --- | --- | --- | --- | --- |
| 1 | 5,6-EET | 4.65 | 1.50E-03 | 0.8819 |
| 2 | 8,9-EET | 3.02 | 1.92E-02 | 0.7813 |
| 3 | 11,12-EET | 8.39 | 1.79E-02 | 0.7847 |
| 4 | 14,15-EET | 3.66 | 1.22E-03 | 0.8889 |
| 5 | 8,9-DHET | 12.43 | 6.58E-04 | 0.9097 |
| 6 | 11,12-DHET | 6.89 | 2.43E-02 | 0.7708 |
| 7 | 6,15-diketo-13,14-dihydro-PGF1a | 3.36 | 5.10E-03 | 0.8368 |
| 8 | 6-keto-PGF1a | 10.13 | 1.67E-02 | 0.7882 |
| 9 | TXB_2_ | 0.17 | 5.10E-03 | 0.8368 |

Fold change (FC) was calculated as the ratio of median lipid level in PQS+DAPT group compared with that in DAPT group. Statistical significance was evaluated by Mann-Whitney *U* test. The value of the area under the curve (AUC) of each lipid was calculated by receiver operating characteristic analysis as shwon in Fig. S2.

**Fig. S2.** Receiver operating characteristic analysis for significantly differential lipids between DAPT group and PQS+DAPT group in plasma (A-D) and gastric mucosa (E-G).

**
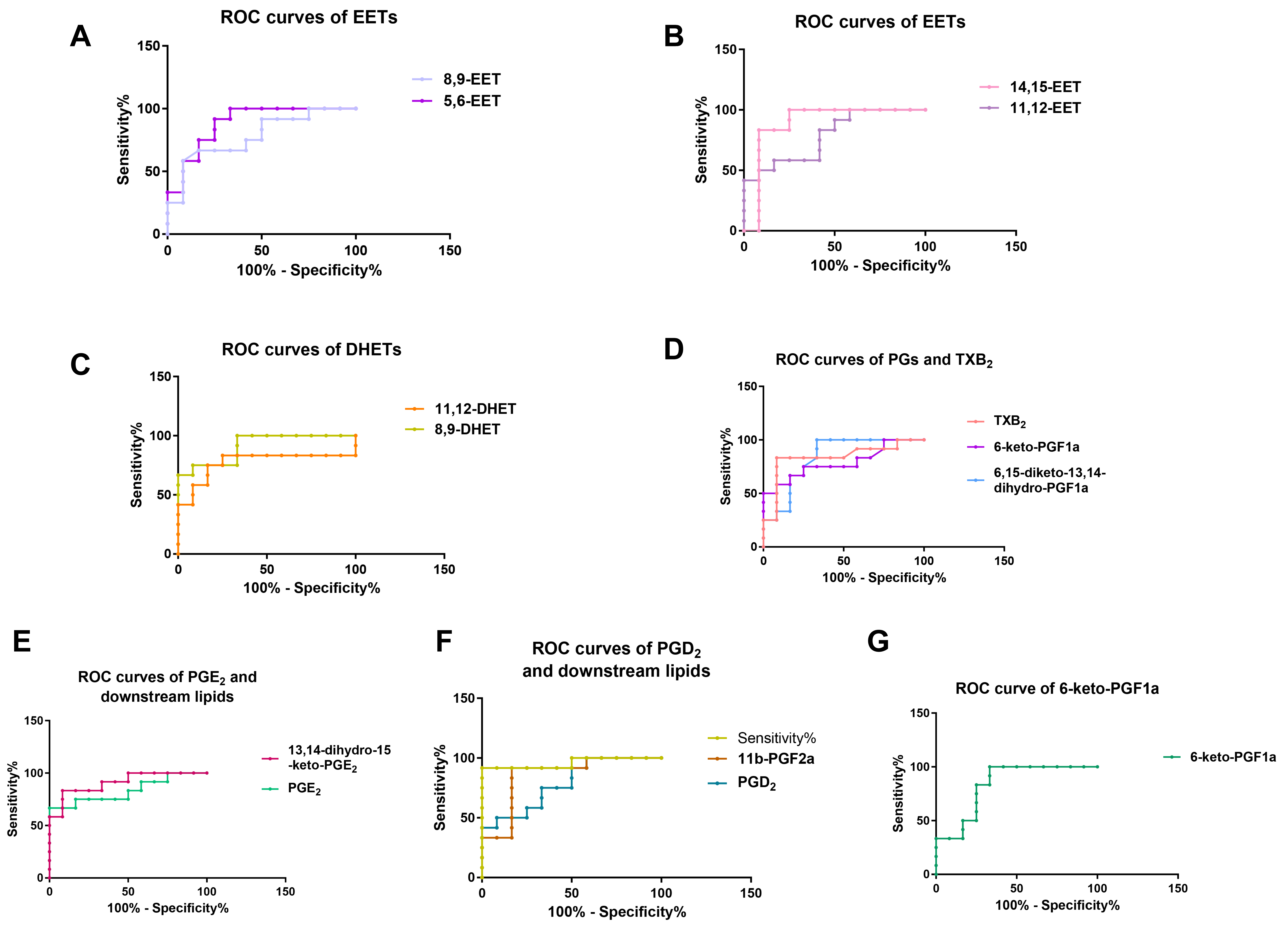
**

**Table S4** AA-derived eicosanoids detected in rat gastric mucosa. Significantly altered eicosanoids between DAPT group and PQS+DAPT group were shown in bold.

| No. | Eicosanoid name | Enzyme | Human Metabolome Database ID | KEGG compound ID |
| --- | --- | --- | --- | --- |
| 1 | **6-keto-PGF1a** | COX | HMDB0002886 | C05961 |
| 2 | 8-iso-PGF2a | free radical-catalyzed | HMDB0005083 | C13809 |
| 3 | TXB_2_ | COX | HMDB0003252 | C05963 |
| 4 | 14,15-DHET | CYP | HMDB0002265 | C14775 |
| 5 | 12-HHT | COX | HMDB0012535 | C20388 |
| 6 | 11,12-DHET | CYP | HMDB0002314 | C14774 |
| 7 | **11b-PGF2a** | COX | HMDB0010199 | C05959 |
| 8 | 8-iso-PGE_2_ | free radical-catalyzed | HMDB0005844 | - |
| 9 | **PGE_2_** | COX | HMDB0001220 | C00584 |
| 10 | **PGD_2_** | COX | HMDB0001403 | C00696 |
| 11 | 13,14-dihydro-15-keto-PGF2a | COX | HMDB0004685 | - |
| 12 | **13,14-dihydro-15-keto-PGE_2_** | COX | HMDB0002776 | C04671 |
| 13 | **13,14-dihydro-15-keto-PGD_2_** | COX | HMDB0060042 | - |
| 14 | LTC_4_ | 5-LOX | HMDB0001198 | C02166 |
| 15 | 11-trans-LTC_4_ | 5-LOX | HMDB0005095 | - |
| 16 | 8,9-DHET | CYP | HMDB0002311 | C14773 |
| 17 | 15-HETE | 15-LOX | HMDB0003876 | C04742 |
| 18 | 9-HETE | free radical-catalyzed | HMDB0010222 | - |
| 19 | 8-HETE | 15-LOX | HMDB0004679 | C14776 |
| 20 | 5-HETE | 5-LOX | HMDB0011134 | C04805 |
| 21 | 11-HETE | COX | HMDB0004682 | C14780 |
| 22 | LTB_4_ | 5-LOX | HMDB0001085 | C02165 |
| 23 | 12-HETE | 12-LOX | HMDB0006111 | C14777 |
| 24 | 14,15-EpETrE | CYP | HMDB0002283 | - |
| 25 | 5,6-EpETrE | CYP | HMDB0002190 | C14768 |
| 26 | AA | - | HMDB0001043 | C00219 |

**Table S5** The information of 7 plasma eicosanoids with VIP higher than 1.

| No. | Eicosanoid name | VIP rank | m/z |
| --- | --- | --- | --- |
| 1 | 13,14-dihydro-15-keto-PGD_2_ | 1.82 | 207.2 |
| 2 | 13,14-dihydro-15-keto-PGE_2_ | 1.58 | 175.1 |
| 3 | 11b-PGF2a | 1.55 | 271.2 |
| 4 | 6-keto-PGF1a | 1.48 | 245.2 |
| 5 | PGE_2_ | 1.47 | 271.2 |
| 6 | PGD_2_ | 1.38 | 271.2 |
| 7 | 11,12-DHET | 1.001 | 167.1 |

Abbreviations: VIP, variable importance in projection; m/z, mass number/charge number.

**Table S6** 6 lipids that were significantly up-/ down-regulated by PQS+DAPT compared with DAPT in rat gastric mucosa.

| No. | Eicosanoid name | FC | *P* value | AUC |
| --- | --- | --- | --- | --- |
| 1 | PGE_2_ | 23.91 | 5.58E-03 | 0.8333 |
| 2 | 13,14-dihydro-15-keto-PGE_2_ | 19.87 | 6.58E-04 | 0.9097 |
| 3 | PGD_2_ | 14.26 | 1.79E-02 | 0.7847 |
| 4 | 11b-PGF2a | 10.06 | 3.24E-03 | 0.8542 |
| 5 | 13,14-dihydro-15-keto-PGD_2_ | 15.76 | 1.39E-04 | 0.9583 |
| 6 | 6-keto-PGF1a | 11.97 | 5.58E-03 | 0.8333 |
